# A Practical Treatise on Materia Medica and Therapeutics, with Especial Reference to the Clinical Application of Drugs

**Published:** 1907-03

**Authors:** 


### BOOK REVIEWS.

A Practical Treatise on Materia Medica and Therapeutics, with Especial Reference to the Clinical Application of Drugs. By John V. Shoemaker, M. D., Professor of Materia Medica, Pharmacology, Therapentics, and Clinical Professor of Diseases of the skin in the Medico-Chirurgical College of Philadelphia Physicion to the Medico-Chirurgical Hospital; Membor of the American Medical Association and the British Medical Association; Fellow of the Medical Society of London, etc., etc. Sixth Edition. Thoroughly Revised. (In Conformity with Latest Revised U. S. Pharmacopoeia, 1905.) Royal Octavo, 1244 Pages. Extra Cloth. Price $5.00 net. Full Sheep. Price, $6.00 net. F. A. Davis Company, Publishers. 1914-16 Cherry Street, Philadelphia, Pa.

This book by Dr. Shoemaker is well written, and is comprehensive. It has three divisions,the first, that on pharmacology, which is essentially an introductory chapter and comprises the first seventy pages; the second division is devoted to drugs, and occupies about eight hundred pages; the third division, which is taken up with a discussion on non pharmacal remedies and expedients, is found between pages 927 and 1158.

The author shows that he is up to date on the recent pharmacologic literature. For example on pages 544 and 545 is found an excellent discussion of the recently discovered chemistry of the alkaloids, atropine, hyoscyanine, hyoscine, atroscine, and scopolamine. Again on page 445 the author presents the latest knowledge on the active chemical constituents of ergot, and gives the pharmacological action of each.

In the third section the author discussed in a succinct intelligent manner practically all the therapeutic measures apart from drugs. Among others he considers the therapeutic application of electricity, massage, water, climate, diet suggestion, temperature, light, music, blood letting, nerve stretching, etc. Tne discussion on all of these topics is ably presented, and embodies a very valuable fund of knowledge for the practical man. We unhesitatingly recommend this book.

